# Sheffield Medical Society, December 12, 1844

**Published:** 1845-04

**Authors:** 


SHEFFIELD MEDICAL SOCIETY, DECEMBER 12, 1844.

*Enlargement of the Heart; Diseased Valves: Hemiplegia.*—Dr. Favell exhibited the heart of a young man, aged 21 years. He was admitted into the Infirmary under the care of Dr. Favell, on Friday, Novembe 8thr. At the time of his admission he complained of slight rheumatic pain in his legs; on visiting him the next morning, he was found hemiplegic; motion and sensation in the right arm and leg were very much impaired; the mouth was drawn to the left side; articulation was very indistinct; and the pupil of the left eye much more dilated than that of the other. He stated that he had a similar attack, but in a much slighter degree, on the previous Sunday. Pulse 90, regular, but vibratile; no pain in the head. On examining the heart, a distinct fremissement was felt when the hand was placed on the præcordial region. The impulse was much increased; the dulness considerably more extensive than natural; and the apex beat on the axillary side of the nipple. A loud rough murmur was heard with the systole at the apex, and also an inch above the ensiform cartilage, and gradually decreased towards the situation of the aortic valves. A softer murmur was also heard along the course of the left ventricle with the diastole. The heart was of twice the natural size, and weighed between sixteen and seventeen ounces; the auricles were much dilated; the right auriculo-ventricular opening dilated; the tricuspid valve opaque, and the free edges indurated; the left auriculo-ventricular opening contracted, so as not to admit the little finger, and furrowed with ossific deposit; the mitral valve short, thick, and cartilaginous.

The walls of the left ventricle very much thickened; the aortic valves rather thicker than natural, but containing no ossific deposit.

*Enlargement of the Breasts in a Male: atrophy of the Genital Organs.*—A man, aged 55, originally a quarryman at Rochester, but afterwards one of the British Legion in Spain, was presented to the Society, having a remarkable development of the breasts. He stated that he had been very healthy, had three children, the eldest 25, the youngest would have been between 8 and 9 years of age; that in an engagement in Spain, he tried to jump over a trench, but, being laden with his knapsack, failed, and fell with his chest against the opposite margin. He received a blow on the lower part of the spine, and falling some depth, received a violent blow on the occiput, which rendered him insensible. He was under the care of Mr. R. Alcock for some time. Since that period the mammæ have gradually increased in size, his voice has diminished in power, his beard has diminished, the right testicle has diminished to a very small size, and the left is still diminishing, and he has had no sexual desire since. He is partially paralysed on the right side, and moves about very much bent. Skin fair and soft; beard very thin and soft; voice weak; mammæ pendulous; nipples rather prominent; the glands very large and distinct; penis small and shrivelled; right testicle having the feeling of condensed membrane; left of a moderate size, but not firm, and tender on pressure; the pelvic region round, more like that of a woman, not presenting the angular appearance of the male. He denies having ever had venereal disease, or taken any quantity of mercury.

Cases were mentioned of enlargement of the breasts in the male, related in the "Philosophical Transactions," vol. 41; and in Captain Franklin's "Journey to the Polar Regions;" and of emasculation consequent upon blows or wounds of the occiput, related by Larrey, in his "Clinique Chirurgicale;" and by Hennen in his "Military Surgery." There was no appearance of abscess in the mammæ.—*Prov. Med. and Surg. Journ.*

